# Longitudinal trajectories of health-related quality of life and their predictors among community-dwelling older adults

**DOI:** 10.1038/s41598-025-30307-8

**Published:** 2025-12-07

**Authors:** Ryoga Oshima, Yuki Ohashi, Takuro Iwane, Yoshinori Tamada, Fumie Kinoshita, Tomoya Ito, Yuto Okumura, Tatsuya Mikami, Ken Itoh, Koichi Murashita, Masahiro Nakatochi

**Affiliations:** 1 Public Health Informatics Unit, Department of Integrated Health Sciences, Nagoya University Graduate School of Medicine, 1-1-20 Daiko-Minami, Higashi-ku, Nagoya, 461-8673 Japan; 2 Research Institute of Health Innovation, Hirosaki University, Hirosaki, Japan; 3 Department of Advanced Medicine, Data Science Division, Data Coordinating Center, Nagoya University Hospital, Nagoya, Japan; 4 Department of Stress Response Science, Biomedical Research Center, Hirosaki University Graduate School of Medicine, Hirosaki, Japan; 5 Department of Medical Data Intelligence, Research Center for Health-Medical Data Science (RCoHMDS), Hirosaki University Graduate School of Medicine, Hirosaki, Japan; 6 Department of Preemptive Medicine, Innovation Center for Health Promotion, Hirosaki University Graduate School of Medicine, Hirosaki, Japan

**Keywords:** Diseases, Health care, Medical research, Psychology, Psychology, Risk factors

## Abstract

The 36-item Short-Form Health Survey (SF-36) is widely used to assess health-related quality of life. However, only few studies have examined longitudinal SF-36 trajectories in general populations, and even fewer have explored their relationships with lifestyle factors, including sleep quality. We conducted a longitudinal analysis of SF-36 subscale trajectories among 910 Japanese individuals aged ≥ 60 years from 2007 to 2018 (4,799 records). Using latent class mixed models, we identified distinct trajectory patterns for each subscale. Notably, the role-physical (RP) and role-emotional (RE) subscales exhibited multiple patterns, including rapid decline and non-decline trajectories. We further examined predictors that differentiated between these two trajectory patterns—rapid decline and non-decline—with similar baseline scores. Consequently, poor performance on the open-eye one-leg standing test and higher depression scores on the Centre for Epidemiologic Studies Depression Scale predicted RP and RE declines, respectively. Importantly, poor sleep quality, as measured by the Pittsburgh Sleep Quality Index—particularly the total score and component 7 (daytime dysfunction)—was associated with subsequent RP and RE declines. In contrast, sleep timing variables (bedtime, sleep onset, and wake-up time) were not predictive. These findings suggest that preserving good sleep quality may help prevent future limitations in daily physical and emotional roles.

**Supplementary Information:**

The online version contains supplementary material available at 10.1038/s41598-025-30307-8.

## Introduction

Globally, population aging due to an increase in life expectancy is a serious problem. The high rate of population aging owing to an increase in welfare economic costs and caregiver shortage has imposed a social burden, significantly impacting the sustainability of the welfare safety net. To reduce the social burden of increased life expectancy, it is essential to maximize healthy life expectancy (HALE). Regarding the global population ageing trend in 2023, Japan had the longest average life expectancy (men: 81.09 years; women: 87.14 years), and the proportion of people aged 65 years and above was 29.1%^1,2^. To improve HALE, the Japanese government has been implementing public health policies to reduce the social burden caused by illness and disability and to prevent serious illness by promoting early intervention (e.g. the National Health Promotion Campaign in the 21 st Century)^3^.

Health-related quality of life (HRQOL), an indicator of HALE, has attracted attention because it comprehensively reflects physical, mental, and social health^4–7^. Recent studies have reported that HRQOL is more than just a subjective health indicator; it predicts health outcomes. For example, low HRQOL scores were reported to be associated with an increased incidence of coronary heart disease (CHD) and mortality^8^. HRQOL can help identify undiagnosed diseases and disease precursors, attracting attention from early disease detection and preventive medicine perspectives. Furthermore, the multifaceted evaluation of HRQOL is also essential for the establishment of a theoretical healthcare foundation because it enables quantitative understanding of not only physical health but also the influence of psychosocial factors and health behaviors^9^.

HRQOL can broadly be used for cross-sectional assessments, which classify individuals as having high and low HRQOL, and longitudinal assessments, which evaluate changes in HRQOL over time^10,11^. To date, many cross-sectional studies have reported associations between HRQOL and lifestyle/medical factors, including sleep^12,13^, exercise^13^, and the social environment^13,14^. In particular, subjective sleep quality, a qualitative factor, may have a greater influence on HRQOL than sleep duration, a quantitative factor, in the sleep patterns of older adults^12^. Longitudinal studies on HRQOL are fewer than cross-sectional studies and have focused on changes in HRQOL in several diseases or pathological conditions^15–19^. Therefore, epidemiological evidence on HRQOL has predominantly been derived from cross-sectional studies, with relatively fewer reports from longitudinal studies observing changes in HRQOL in the general population. Consequently, predictors of HRQOL trajectories, especially those of nonspecific diseases, remain largely unclarified. Given the growing evidence from cross-sectional studies linking sleep, depressive symptoms, and physical function to HRQOL and the fact that these factors can be assessed in general populations, this study specifically selected these factors as potential predictors. Among them, sleep is particularly important, as it has been consistently associated with HRQOL across multiple studies and represents a potentially modifiable factor that could prevent future declines in HRQOL.

Accordingly, this longitudinal study aimed to investigate the trajectories and predictors of HRQOL among community-dwelling older adults. This study can help identify predictors of HRQOL decline, thereby enabling early intervention. Given the central role of sleep highlighted in prior research, we examined its association with HRQOL in detail. To the best of our knowledge, this is the first large longitudinal study in Japanese community-dwelling older adults to explore HRQOL trajectories, with the novel approach of simultaneously modelling multiple SF-36 subscales and examining sleep quality as a potential predictor.

## Methods

### Study design and participants

**Fig. 1:**
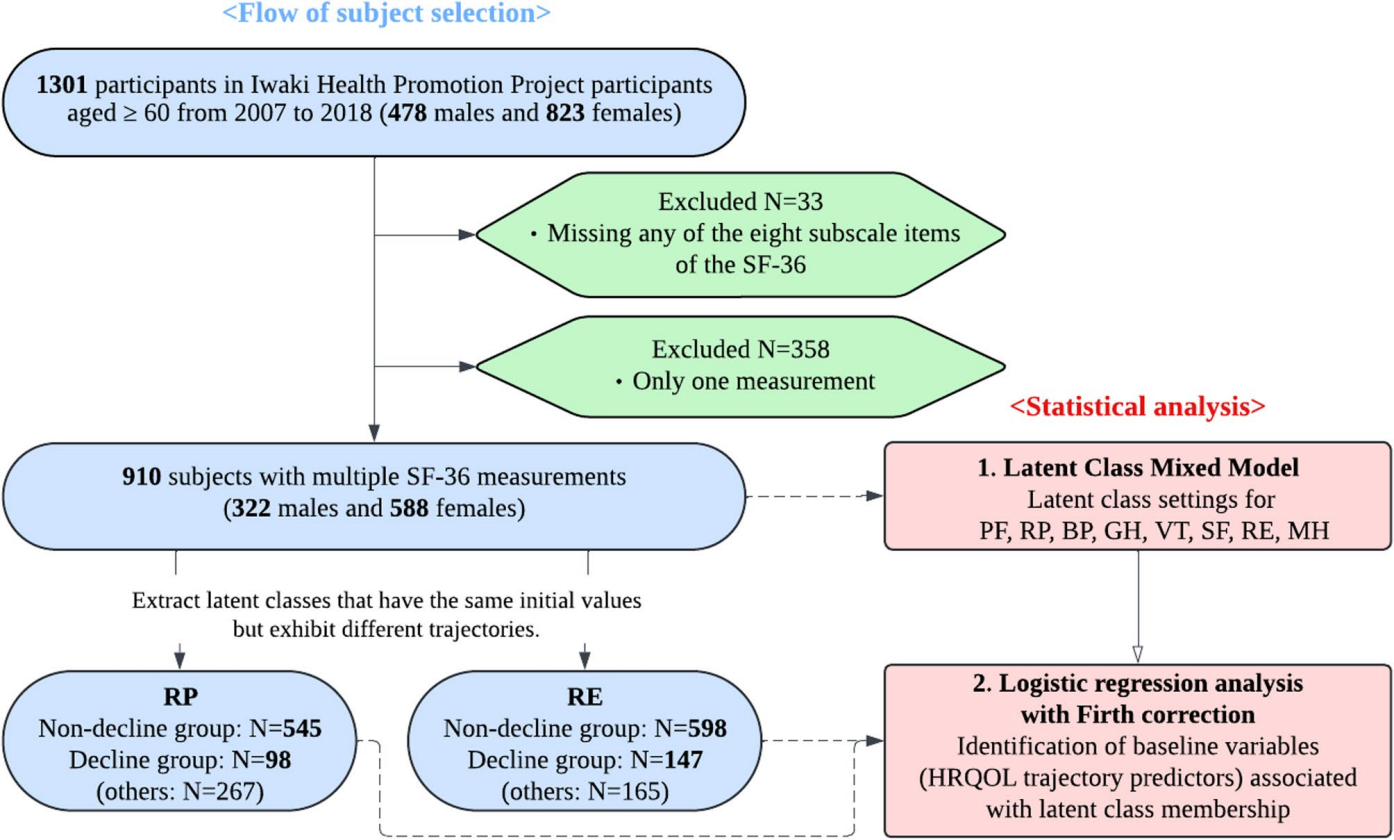
Flow diagram of the longitudinal study design. Abbreviations: PF, physical functioning; RP, role-physical; BP, bodily pain; GH, general health; VT, vitality; SF, social functioning; RE, role-emotional; MH, mental health.

This longitudinal study included residents in the Tohoku region of Japan. Data were collected on the health information of residents, obtained through the “Iwaki Health Promotion Project” in the Iwaki area of Hirosaki city, Aomori prefecture, initiated by Hirosaki University^20–22^, which has been recording longitudinal HRQOL data since April 2007. In addition to HRQOL data, we collected data on clinical parameters, sleep quality, and mental health (see sub-section “Data collection” for details) from 2007 to 2018, immediately before the coronavirus disease 2019 pandemic. A total of 1,301 participants in the Iwaki Health Promotion Project between 2007 and 2018 aged ≥ 60 years were included in this study. To evaluate the HRQOL trajectory within the observational period, participants with missing data (*n* = 33) or only one HRQOL measurement (*n* = 358) were excluded, leaving 910 participants, with a total of 4,799 records for each SF-36 subscale, for analysis (Fig. 1).

### Ethics statement

This study was conducted in accordance with the Declaration of Helsinki and approved by the Medicine Ethics Review Committee of Hirosaki University (approval #2024-060) and Nagoya University (approval #2024-0180). All participants provided written informed consent. This study was conducted as a secondary analysis using data from participants who voluntarily participated in the Iwaki Health Promotion Project.

### Public involvement

Participants were not involved in the development of the research questions or outcome measures, nor were participants involved in the development of the study’s implementation plan. No participants were asked for advice on the interpretation or writing of the results.

### Data collection

#### Clinical, laboratory, and lifestyle variables

In the Iwaki Health Promotion Project, multi-item physical and questionnaire surveys were conducted to determine the overall condition of the whole body. Body mass index, calculated as weight (kg)/height squared (m^2^), body fat percentage, the open-eye one-leg standing test, and grip test were assessed. The questionnaire surveyed whether the participants currently use alcohol, exercise, or smoke tobacco. Educational history (more than 12 years) and family structure were also investigated.

#### HRQOL variables

We used the Japanese version of the 36-item Short-Form Health Survey (SF-36) to quantitatively assess HRQOL (License Number: A-05897–250004)^23,24^. The SF-36 is one of the most utilized self-report health-related scales worldwide and is a comprehensive quality of life (QOL) scale that is not limited to specific diseases or targets. The SF-36 measures eight health concepts: physical functioning (PF), role-physical (RP), bodily pain (BP), general health (GH), vitality (VT), social functioning (SF), role-emotional (RE), and mental health (MH). It is a 100-point scale, with higher scores indicating higher HRQOL in each concept^23,24^.

#### Sleep quality variables

The Japanese version of the Pittsburgh Sleep Quality Index (PSQI) was used to assess sleep. It consists of seven components: subjective sleep quality (C1), sleep latency (C2), sleep duration (C3), habitual sleep efficiency (C4), sleep disturbances (C5), sleep medication use (C6), and daytime dysfunction (C7). The PSQI is scored per subscale and by the total score^25,26^. In addition to the PSQI scores, we recorded the time the participants went to bed, their sleep onset time, and their wake-up time using a self-administered questionnaire at baseline.

#### Mental health variables

The Centre for Epidemiologic Studies Depression Scale (CES-D) was used to assess depressive symptoms. The CES-D is a 60-point self-rating questionnaire that assesses depressive symptoms over the past week. Based on previous cutoff values, a score of 16 or higher was considered a depressive tendency, and the scores were treated as binary categorical data^27^.

### Statistical analysis

We summarized continuous and categorical data of baseline participant characteristics, and data are presented as median (interquartile range [IQR]) or count (percentage), respectively. To compare the distributions of continuous and categorical variables between men and women at baseline, we used the Wilcoxon rank sum and Fisher’s exact probability tests, respectively.

To establish latent classes by modelling the longitudinal trajectories of the HRQOL, we used latent class mixed models (LCMMs) with the longitudinal data of each of the eight SF-36 subscales^28^. The utility of LCMM for trajectory analysis has been demonstrated in previous research; a study used LCMMs to examine the trajectories of depressive symptoms^29^. A total of 4,799 records of multiple SF-36 scores from 910 participants were used to model the predicted trajectories of SF-36 scores. Fixed effects were defined as the number of years that had elapsed since the initial assessment, age at the initial assessment, and sex while random effects were defined as the number of years that had elapsed since the initial assessment. Model selection was conducted in two steps. For the first step, the best among three model types was selected: LCMMs with linear, beta, and spline link functions with five knots placed in percentiles. Convergence was evaluated based on three convergence criteria (parameter stability, log-likelihood stability, and derivatives), and only models that successfully converged (all elements of the gconv vector < 1 × 10^− 4^) were considered. Among the converged models, those with the lowest Akaike information criterion (AIC) were selected for each subscale. For the second step, the optimal number of latent classes was determined for the selected model type. We constructed candidate models with latent class numbers ranging from one to five. Among the converged models, we excluded models containing latent classes with fewer than 5% of participants to avoid model overfitting, following the recommendation that each class should include at least 5% of the sample^30^. From the remaining models, the one with the lowest AIC was selected as the optimal model, while the Bayesian information criterion (BIC) and entropy values were reported as supplementary indicators for reference. The HRQOL trajectory for each latent class based on the optimal model was plotted as the number of years elapsed from the mean age at baseline.

Logistic regression was used to identify predictors of latent classes based on the pattern of SF-36 trajectories. In cases where multiple latent classes were present and their baseline SF-36 subscale scores were approximately equivalent, logistic regression analysis was conducted according to the HRQOL decline and non-decline groups; latent classes with baseline SF-36 subscale scores close to each other were considered as dependent variables (reference: non-decline group), and the health assessment item scores at the initial assessment were used as independent variables. Logistic regression with Firth correction was used to account for cases in which the maximum likelihood estimators of the logistic regression analysis did not converge because of the expected imbalance among categories due to the small number of people in the decline group compared to the non-decline group or due to the risk of quasi-complete separation in certain medical examination items^31^. Age, sex, and each baseline SF-36 subscale score were adjusted. In addition, because the baseline RP and RE scores of the decline and non-decline groups were not completely consistent, a subgroup analysis was conducted as a sensitivity analysis to evaluate potential overadjustment bias of the baseline RP and RE scores, limiting the analysis to only participants with a baseline score of 100 in the RP and RE without adjusting for RP and RE scores. No correction for multiple testing was applied because each hypothesis was examined independently, consistent with the rationale for *individual testing* described by Rubin^32^. In this framework, correction for multiple comparisons is not required when each hypothesis addresses a distinct and theoretically motivated research question.

The mean values and distribution of sleeping, falling asleep, and waking times were examined by comparing the non-decline and decline groups. A circular general linear model based on the Markov chain Monte Carlo algorithm, adjusted for age and sex, was used for the comparison. Variables were considered effective if at least one of the 95% highest probability density (HPD) intervals of the linear coefficients for components I and II did not contain 0.

Statistical analysis was performed using R (ver. 4.5.1; R Foundation for Statistical Computing, Vienna, Austria; https://www.r-project.org/)^33^. The ‘lcmm’ package was used to perform the LCMM analysis, and the ‘logistf’ package was used for the logistic regression analysis with Firth correction^28,34^. The ‘circular’ package was used for the calculation and to assess the distribution of mean sleep time, and the ‘bpnreg’ package was used for comparisons^35,36^. The significance level was set at 5% for all the analyses.

## Results

### Summary of baseline participant characteristics

A total of 4,799 records of SF-36 scores were obtained from 910 participants in this study, and the age distribution is shown in Supplementary Figure S1a. Of the 910 participants in this study, 116 (12.7%) had SF-36 score data for 10 years (Supplementary Figure S1b). Descriptive statistics by the sex of the participants are shown in Table 1; 322 (35.4%) and 588 (64.6%) were men and women, respectively. The median age was 64.0 (IQR: 60.3–70.0) years for men and 64.0 (IQR: 60.0–70.0) years for women, with no significant difference (*p* = 0.314). The median body mass index for men (23.6 [IQR: 21.9–25.6] kg/m^2^) was significantly higher (*p* = 0.007) than that for women (23.0 [IQR: 21.2–25.0] kg/m^2^), and the median body fat percentage for men (20.7% [IQR: 16.7–24.5%]) was significantly lower (*p* < 0.001) than that for women (31.2% [IQR: 27.0–35.3%])). The SF-36 subscale scores (PF, RP, BP, GH, VT, and RE) were significantly higher in men than in women (PF: *p* < 0.001, RP: *p* = 0.001, BP: *p* = 0.002, GH: *p* = 0.003, VT: *p* = 0.002, RE: *p* = 0.042), with no significant differences observed for SF (*p* = 0.058) and MH (*p* = 0.210) between sexes.

**Table 1:** Baseline characteristics of participants and comparison between men and women.

	Women	Men	
	*N* = 588	*N* = 322	*p*-value*
Age (years)	64.0 (60.0–70.0)	64.0 (60.3–70.0)	0.314
Body mass index (kg/m^2^)	23.0 (21.2–25.0)	23.6 (21.9–25.6)	0.007
Body fat percentage (%)	31.2 (27.0–35.3)	20.7 (16.7–24.5)	< 0.001
Length of education			
> 12 years	64 (11.1%)	47 (14.7%)	0.134
≤ 12 years	524 (88.9%)	275 (85.3%)	
SF-36 subscale score			
Physical functioning	85.0 (70.0–95.0)	90.0 (85.0–95.0)	< 0.001
Role-physical	100.0 (75.0–100.0)	100.0 (82.8–100.0)	0.001
Bodily pain	72.0 (52.0–100.0)	74.0 (62.0–100.0)	0.002
General health	57.0 (50.0–72.0)	62.0 (52.0–72.0)	0.003
Vitality	68.8 (50.0–81.3)	75.0 (56.3–81.3)	0.002
Social functioning	100.0 (87.5–100.0)	100.0 (87.5–100.0)	0.058
Role emotional	100.0 (83.3–100.0)	100.0 (91.7–100.0)	0.042
Mental health	80.0 (65.0–90.0)	80.0 (65.0–90.0)	0.210
Drinking habits			
Never or past drinking	482 (82.0%)	91 (28.0%)	< 0.001
Current drinking	106 (18.0%)	231 (72.0%)	
Exercise habits			
≥ 1 time per week	187 (31.8%)	11 (34.5%)	0.455
< 1 time per week	401 (68.2%)	311 (65.5%)	
Smoking habit			
Never or past smoking	564 (95.9%)	243 (75.5%)	< 0.001
Current smoking	24 (4.1%)	79 (24.5%)	
Current marital status			
Yes	413 (68.6%)	290 (89.7%)	< 0.001
No	175 (31.4%)	32 (10.3%)	
Family structure	3.0 (2.0–5.0)	3.0 (2.0–5.0)	0.326
Open-eye one-leg standing (sec)	7.0 (2.9–7.0)	8.0 (3.8–8.0)	< 0.001
Grip strength (kg)	25.0 (22.5–28.0)	39.8 (35.0–44.0)	< 0.001
MMSE score	29.0 (28.0–30.0)	29.0 (27.0–30.0)	< 0.001
CES-D score			
≥ 16 point	492 (81.0%)	291 (89.2%)	0.004
< 16 point	96 (19.0%)	31 (10.8%)	
Total PSQI score	3.0 (2.0–5.0)	2.0 (1.0–4.0)	< 0.001
C1: subjective sleep quality	1.0 (0.0–1.0)	1.0 (0.0–1.0)	0.377
C2: sleep latency	0.0 (0.0–1.0)	0.0 (0.0–1.0)	< 0.001
C3: sleep duration	1.0 (0.0–1.0)	0.0 (0.0–1.0)	< 0.001
C4: habitual sleep efficiency	0.0 (0.0–0.0)	0.0 (0.0–0.0)	0.459
C5: sleep disturbances	1.0 (0.0–1.0)	1.0 (0.0–1.0)	0.827
C6: use of sleeping medication	0.0 (0.0–0.0)	0.0 (0.0–0.0)	< 0.001
C7: daytime dysfunction	0.0 (0.0–1.0)	0.0 (0.0–1.0)	0.046

Values are presented as median (interquartile range) and count (%). *p-values were calculated using Wilcoxon rank sum test or Fisher’s exact test. SF-36, 36-item Short-Form Health Survey; MMSE, Mini-Mental State Examination; CES-D, Centre for Epidemiologic Studies Depression Scale; PSQI, Pittsburgh Sleep Quality Index.

### Classification based on the trajectory of RP and RE SF-36 subscale scores during the study period.

To model the longitudinal trajectories of HRQOL and establish latent classes, we employed LCMMs using longitudinal data for each of the eight SF-36 subscales. As shown in Supplementary Table S1, we compared LCMMs with linear, beta, and spline link functions, with five knots placed in percentiles for each SF-36 subscale to determine the best-fitting approach. LCMMs with beta link function provided the best fit based on the AIC for four subscales, including the PF, BP, VT, and MH subscales, while spline link function with five knots placed in percentiles was selected for the RP, SF, RE, and GH subscales. Using these best-fitting models, the optimal number of latent classes was selected (Supplementary Table S2). Consequently, we identified three classes for RP and RE, two classes for PF, BP, VT, SF, and MH, and one class for GH.

**Fig. 2:**
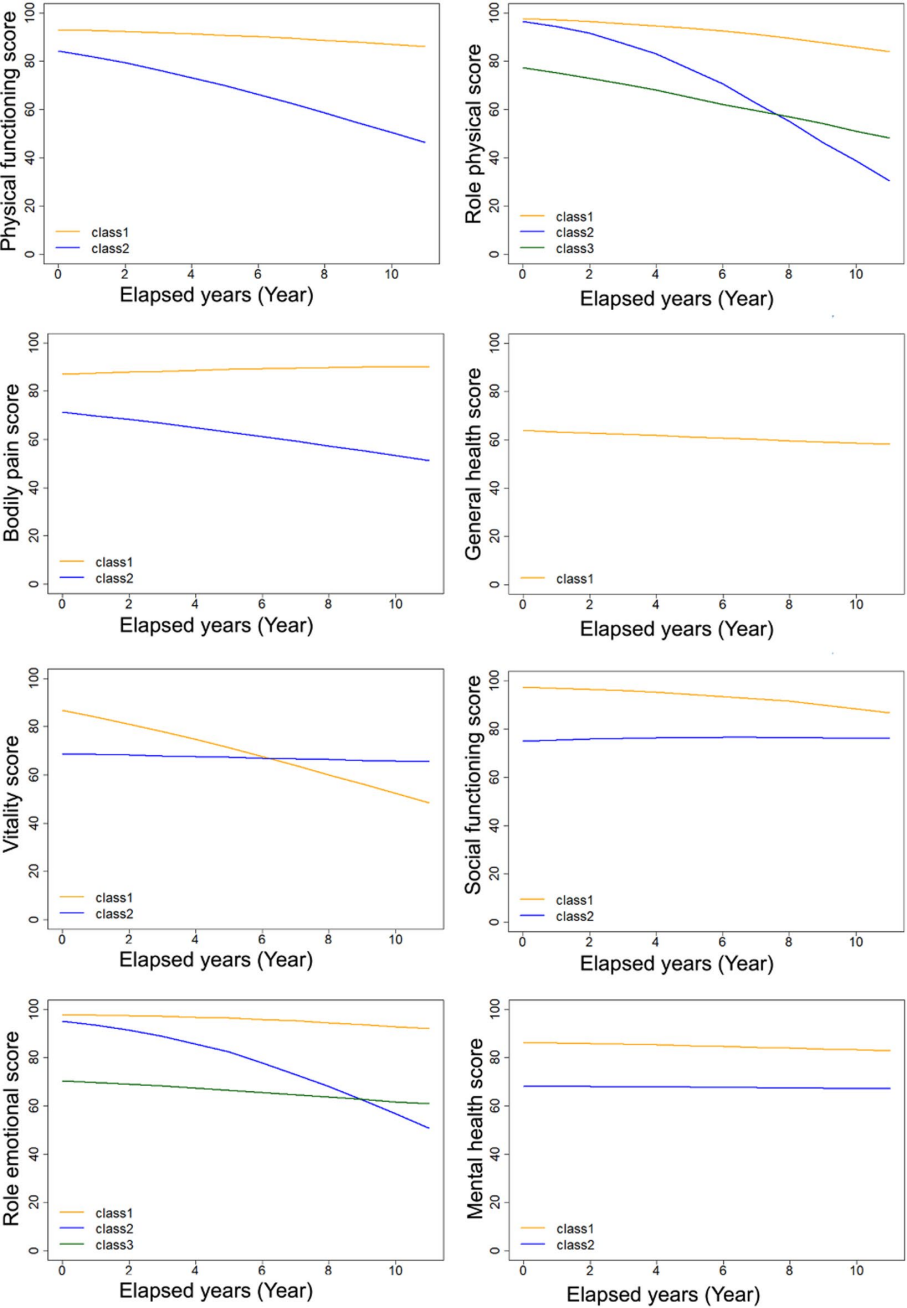
Each SF-36 subscale trajectory pattern by latent class mixed models. The trajectories of the eight subscales of the SF-36 from the latent class mixture model are shown. Sex is fixed at men and age at the baseline mean age of 65.6 years.

As shown in Fig. 2, we identified the trajectories of all the subscale scores, except the GH score, into several transition patterns over time. We identified the participants into three latent classes based on different RP score trajectory patterns (Supplementary Table S3), three latent classes based on different RE score trajectory patterns (Supplementary Table S4), and two latent classes based on each of the PF, BP, VT, SF, and MH score trajectory patterns in this longitudinal study. In these three RP and RE trajectory patterns, we particularly focused on latent classes that had similar scores at baseline but different trajectories. A latent class maintained high scores (class 1, defined as the ‘non-decline group’), and another had rapidly declining scores (class 2, defined as the ‘decline group’), although their scores at baseline were almost equally high (Fig. 2). Regarding the RP score, the non-decline and decline groups had 545 and 98 participants, with baseline median scores of 100.0 (IQR: 100.0–100.0) and 100.0 (IQR: 93.8–100.0), respectively. Regarding the RE score, the non-decline and decline groups had 598 and 147 participants, with baseline median scores of 100.0 (IQR: 100.0–100.0) and 100.0 (IQR: 87.5–100.0), respectively. We have summarized the baseline characteristics of participants classified into the non-decline and decline groups based on the RP and RE in Table 2.

**Table 2:** Summary of characteristics by latent classes based on the longitudinal trajectories of the role-physical and role-emotional SF-36 subscale scores at baseline.

	Role-physical		Role-emotional	
	Class 1: Non-decline group	Class 2: Decline group	Class 1: Non-decline group	Class 2: Decline group
	*N* = 545	*N* = 98	*N* = 598	*N* = 147
Age (years)	63.0 (60.0–68.0)	64.5 (60.0–72.0)	63.0 (60.0–68.0)	65.0 (60.0–71.5)
Men	207 (38.0%)	33 (33.7%)	221 (37.0%)	56 (38.1%)
Body mass index (kg/m^2^)	23.1 (21.2–25.0)	23.5 (21.7–25.6)	23.1 (21.3–25.1)	23.6 (21.8–25.8)
Body fat percentage (%)	26.4 (21.0–31.6)	28.5 (22.4–33.2)	26.5 (21.2–32.2)	28.1 (23.3–32.2)
Length of education				
> 12 years	70 (13.0%)	9 (9.4%)	77 (13.0%)	14 (9.7%)
≤ 12 years	475 (87.0%)	89 (90.6%)	521 (87.0%)	133 (90.3%)
SF-36 subscale scores				
Physical functioning	90.0 (85.0–95.0)	90.0 (75.0–90.0)	90.0 (80.0–95.0)	85.0 (70.0–95.0)
Role-physical	100.0 (100.0–100.0)	100.0 (93.8–100.0)	100.0 (93.8–100.0)	93.8 (75.0–100.0)
Bodily pain	84.0 (62.0–100.0)	72.0 (62.0–84.0)	74.0 (62.0–100.0)	72.0 (57.5–84.0)
General health	62.0 (52.0–77.0)	56.0 (52.0–67.0)	62.0 (52.0–76.5)	57.0 (51.0–72.0)
Vitality	75.0 (62.5–87.5)	68.8 (56.3–87.5)	75.0 (62.5–95.0)	62.5 (50.0–75.0)
Social functioning	100.0 (100.0–100.0)	100.0 (87.5–100.0)	100.0 (100.0–100.0)	100.0 (81.3–100.0)
Role-emotional	100.0 (100.0–100.0)	100.0 (93.8–100.0)	100.0 (100.0–100.0)	100.0 (87.5–100.0)
Mental health	85.0 (70.0–95.0)	80.0 (65.0–90.0)	85.0 (70.0–93.8)	75.0 (60.0–90.0)

Values are presented as median (interquartile range) and count (%). SF-36, 36-item Short-Form Health Survey.

### Exploratory analysis of the predictors that distinguish between future decline and non-decline groups based on the RP and RE scores

We performed Firth’s logistic regression analysis to explore baseline measurements that distinguish the RP score decline group from the non-decline group (Table 3). In the RP decline group, for the physical assessment, current exercise habits of once a week or more was significantly associated with a reduced risk of RP decline (odds ratio [OR] = 0.60, 95% confidence interval [CI] = 0.36–0.98, *p* = 0.041), and better performance on the open-eye one-leg standing test was significantly associated with a reduced risk of RP decline (OR = 0.89, 95% CI = 0.80–0.98, *p* = 0.019). For sleep assessments, a higher total PSQI score was significantly associated with an increased risk of RP decline (OR = 1.12, 95% CI = 1.02–1.23, *p* = 0.020), as was the PSQI C2 score (OR = 1.34, 95% CI = 1.02–1.74, *p* = 0.036). In particular, the PSQI C7 score was significantly associated with an increased risk of RP decline (OR = 1.87, 95% CI = 1.30–2.67, *p* < 0.001).

**Table 3:** The Firth logistic regression analysis results identifying predictors of the SF-36 role-physical/role-emotional decline and non-decline groups.

	Role-physical			Role-emotional		
	Complete data *n* (%)	OR (95% CI)	*p*-value	Complete data *n* (%)	OR (95% CI)	*p*-value
Body mass index category	643 (100%)			745 (100%)		
Normal range (18.5–25.0 kg/m^2^)		1 (reference)			1 (reference)	
Overweight/obesity (> 25 kg/m^2^)		1.44 (0.89–2.30)	0.139		1.52 (1.01–2.26)	0.044
Underweight (≤ 18.5 kg/m^2^)		0.70 (0.22–1.77)	0.478		0.77 (0.29–1.76)	0.550
Body fat percentage	643 (100%)	1.03 (0.99–1.07)	0.060	745 (100%)	1.03 (0.99–1.06)	0.103
Lifestyles						
Presence of drinking habits	642 (99.8%)	0.76 (0.45–1.28)	0.313	744 (99.9%)	0.84 (0.53–1.32)	0.458
Presence of exercise habits	643 (100%)	0.60 (0.36–0.98)	0.041	745 (100%)	1.02 (0.69–1.52)	0.906
Presence of smoking habit	643 (100%)	1.05 (0.48–2.12)	0.888	745 (100%)	0.85 (0.43–1.57)	0.607
Length of education	636 (98.9%)	0.81 (0.37–1.60)	0.564	737 (98.9%)	0.84 (0.44–1.50)	0.771
Household structure						
Marital status	622 (96.7%)	1.13 (0.65–1.91)	0.666	714 (95.8%)	0.87 (0.52–1.40)	0.560
Family structure	642 (99.8%)	1.04 (0.92–1.16)	0.564	744 (99.9%)	1.01 (0.91–1.11)	0.896
Open-eye one-leg standing (10 s)	554 (86.2%)	0.89 (0.80–0.98)	0.019	639 (85.8%)	0.94 (0.86–1.02)	0.146
Grip strength (kg)	558 (86.8%)	0.98 (0.93–1.03)	0.397	645 (86.6%)	0.99 (0.95–1.03)	0.555
MMSE score	417 (64.9%)	0.94 (0.83–1.07)	0.350	474 (63.6%)	0.96 (0.86–1.08)	0.485
CES-D score	553 (86.0%)	1.52 (0.70–3.05)	0.278	645 (86.5%)	2.09 (1.13–3.76)	0.019
Total PSQI score	632 (98.3%)	1.12 (1.02–1.23)	0.020	733 (98.4%)	1.19 (1.09–1.29)	< 0.001
C1: subjective sleep quality	640 (99.5%)	1.09 (0.76–1.55)	0.627	742 (99.6%)	1.27 (0.94–1.73)	0.119
C2: sleep latency	638 (99.2%)	1.34 (1.02–1.74)	0.036	740 (99.3%)	1.18 (0.92–1.50)	0.181
C3: sleep duration	643 (100%)	1.07 (0.80–1.44)	0.620	745 (100%)	1.34 (1.04–1.72)	0.025
C4: habitual sleep efficiency	643 (100%)	1.13 (0.62–1.84)	0.662	745 (100%)	0.95 (0.53–1.57)	0.849
C5: sleep disturbances	638 (99.2%)	1.25 (0.83–1.89)	0.279	740 (99.3%)	1.02 (0.72–1.44)	0.915
C6: use of sleeping medication	641 (99.7%)	1.14 (0.79–1.57)	0.454	743 (99.7%)	1.69 (1.30–2.19)	< 0.001
C7: daytime dysfunction	639 (99.3%)	1.87 (1.30–2.67)	< 0.001	740 (99.3%)	2.05 (1.50–2.82)	< 0.001

Odds ratios (ORs), 95% confidence intervals (CIs), and *p*-values are adjusted for age, sex, and each baseline SF-36 subscale score. Centre for Epidemiologic Studies Depression Scale (CES-D) of 16 or higher was considered a depressive tendency, and the scores were treated as binary categorical data (CES-D ≥ 16 as 1; CES-D < 16 as 0). MMSE, Mini-Mental State Examination; PSQI, Pittsburgh Sleep Quality Index.

The Firth logistic regression analysis results for distinguishing the RE decline group from the non-decline group are shown in Table 3. In the RE decline group, being overweight compared to having a normal weight was significantly associated with an increased risk of RE decline (OR = 1.52, 95% CI = 1.01–2.26, *p* = 0.044), and a higher CES-D score was significantly associated with a greater risk of RE decline (OR = 2.09, 95% CI = 1.13–3.76, *p* = 0.019). Similarly to in the analysis of RP trajectories, a higher total PSQI score was significantly associated with an increased risk of RE decline (OR = 1.19, 95% CI = 1.09–1.29, *p* < 0.001). The component of the PSQI that was most strongly associated with the RE decline was C7 (OR = 2.05, 95% CI = 1.50–2.82, *p* < 0.001), but C6 (OR = 1.69, 95% CI = 1.30–2.19, *p* < 0.001), and C3 (OR = 1.34, 95% CI = 1.04–1.72, *p* = 0.025) were also statistically significantly associated with an increased risk of RE decline.

The sensitivity analysis results, restricted to only participants with baseline scores of 100 in each of the RP and RE subscale scores, are shown in Supplementary Tables S5. In the RP decline group, significant associations remained for current exercise habits, the open-eye one-leg standing test, the total PSQI score, and the PSQI C7 scores, while no significant association remained for the PSQI C2 score. In addition, a new significant association was observed with body fat percentage (Supplementary Table S5). In the RE decline group, significant associations remained for all measurements except the PSQI C3 score (Supplementary Table S5).

### Additional analysis of the association between the RP and RE trajectories and sleep deration

Analyses of bedtime, sleep onset time, and wake-up time were performed to additionally investigate the sleep condition related to the decline and non-decline groups of both the RP and RE scores. The histograms for bedtime, sleep onset time, and wake-up time for each hour of the day are shown in Supplementary Figure S2. The circular generalized linear model results showed that the HPD intervals for bedtime, sleep onset, and wake-up time contained zero. Between the RP non-decline and decline groups, mean bedtime (22:00 and 21:52), sleep onset (22:13 and 22:08), and wake-up time (5:24 and 5:30) were almost equivalent. Similarly, between the RE non-decline and decline groups, no difference in mean bedtime (21:59 and 21:54), sleep onset (22:13 and 22:08), and wake-up time (5:25 and 5:22) was observed.

## Discussion

The present longitudinal study observed longitudinal trajectories of the SF-36 scores of Japanese community-dwelling older adults and demonstrated that distinct patterns of the longitudinal trajectories exist for each subscale of the SF-36 scale. Our longitudinal study revealed for the first time the existence of several patterns in the longitudinal trajectories of all SF-36 subscale scores, except the GH score. These distinct patterns suggest that HRQOL does not decline uniformly with increasing age. In particular, for certain subscales such as RP and RE, we identified specific factors that explain differences in decline patterns between groups.

Among the longitudinal trajectories of the SF-36 subscale scores identified in our study, the longitudinal observations of the RP and RE scores particularly revealed intriguing trajectory patterns. Notably, despite the high baseline RP and RE scores, some participants exhibited a rapid declining pattern, while others maintained stable scores over time. Therefore, our findings imply that the high RP and RE baseline scores did not necessarily predict future trajectories, highlighting the uncertainty in predicting RP and RE trajectory patterns based on only baseline measurements. Therefore, we explored potential predictive factors among baseline measurements other than the RP and RE that could distinguish future RP and RE trajectory patterns. Regarding the RP trajectory, baseline high body fat percentage, high PSQI scores, and poor open-eye one-leg standing test performance, which assess musculoskeletal instability and fall risk^37–40^, were associated with future RP score decline when the baseline RP scores were high. The open-eye one-leg standing test does not require special equipment and can be performed within a short time. The test may not only be useful for screening fall risk but also for predicting future declines in the physical aspect of QOL in daily life functioning. Regarding the RE trajectory analysis, baseline overweight, high PSQI scores, and high CES-D scores were associated with future RE score decline when the baseline RE scores were high. Previous cross-sectional studies have reported an association between depressive tendencies and HRQOL^13,41,42^. Our study suggests that, in addition to the previously reported correlation between depression and HRQOL, low CES-D scores may precede declines in emotional QOL related to daily functioning.

Notably, high PSQI scores indicating sleep disturbances were commonly associated with declines in both RP and RE scores: RP decline was associated with the total PSQI score and their C7 (daytime dysfunction) score, and RE decline was associated with the total PSQI score and their C1 (subjective sleep quality), C6 (use of sleeping medication), and C7 scores. Our longitudinal study suggests that self-reported PSQI scores may change prior to self-reported SF-36 RP and RE subscale scores. Among the PSQI component scores, daytime dysfunction including daytime sleepiness was clearly associated with declines in both RP and RE scores in this study. Previous studies have reported that chronic sleep disturbances has a cumulative effect on physical health, leading to reduced activity participation and activity difficulties^43^. In particular, daytime sleepiness is an essential manifestation of sleep disturbances or disorders and affects an individual’s performance socially, academically, and occupationally^44^. To date, although the primary concern related to daytime sleepiness has often focused on accidents caused by drowsy driving^45,46^, our findings suggest that the impact of daytime sleepiness extends beyond traffic accidents, affecting HRQOL. Additionally, we investigated sleep schedules (bedtime, sleep onset time, and wake-up time) between the groups with and without declines in RP and RE scores, using circular generalized linear model analysis based on the Markov chain Monte Carlo algorithm; however, the mean bedtime, sleep onset time, and wake-up time values were almost similar between the groups, indicating no significant differences in sleep schedules throughout the day. This may implicitly suggest that sleep quality, rather than sleep time and wakefulness associated with lifestyle choices, is extremely important. Poor sleep quality is often accompanied by underlying conditions that can be detected through medical screening, allowing for early intervention. An example of such conditions related to poor sleep quality is obstructive sleep apnea (OSA), which is known for its high prevalence (approximately 20% in men and 10% in women)^47^. Furthermore, in our study, high body fat percentage was associated with RP decline, whereas overweight status was significantly associated with decline in RE score, and it is a known risk factor for OSA^48^. Our findings, which demonstrated that sleep disturbances can predict future RP and RE scores, may contribute to the evidence supporting the additional benefits to current medical strategies aimed at promoting high-quality sleep.

From a prevention and community-care perspective, a pragmatic stepped-care pathway may help maintain HRQOL. As a low-intensity first step, sleep-hygiene education—delivered in primary care or public-health programs—can be offered^49^. For individuals with persistent symptoms or functional impairment, escalation to evidence-based behavioral therapies, such as cognitive behavioral therapy for insomnia, is warranted^50^. In parallel, given the substantial global burden of OSA, risk-based screening in community settings, with referral for diagnostic evaluation and treatment, represents a realistic and high-yield strategy to protect HRQOL in those at risk^51^. These practical pathways provide a framework for feasible preventive and clinical strategies for maintaining HRQOL in community settings.

### Study strengths

The strength of this study lies in the fact that we collected HRQOL subscale scores longitudinally and obtained comprehensive baseline information on physical, mental, and sleep quality assessments including data on open-eye one-leg standing test, CES-D scores, and PSQI scores. The longitudinal measurements of HRQOL enabled flexible classification of latent longitudinal trajectory patterns using LCMM analysis, accounting for nonlinearity. Additionally, comprehensive information enabled the prediction of future HRQOL trajectories related to daily functioning, which are indistinguishable at baseline. Since the predictive factors we identified are based on non-invasive measurements and self-reported questionnaires, researchers can aim for a practical application that minimizes participants’ physical discomfort and potential costs.

### Limitations

Several limitations of the present study must be acknowledged. First, this study was not a retrospective cohort study but an annual panel data analysis, and the tracking of participants’ SF-36 scores was not complete. Therefore, it does not fully reflect the HRQOL trajectory of all participants. In addition, the unmeasured environmental and social changes resulting from the long-term nature of the study cannot be ruled out. Second, this study included participants from only one region, and participation was voluntary. Therefore, participant selection may have been biased towards individuals with a greater interest in health promotion compared with that observed in the general population. The Iwaki Health Promotion Project, from which this study population was drawn, was established as a platform for social innovation through collaboration among local governments, universities, and communities, aiming to promote health equity and extend healthy life expectancy^20^. This community-based and participatory framework reflects social structures unique to the Iwaki area, such as high civic engagement and organized municipal health initiatives. Therefore, while caution is warranted when generalizing the findings to the broader Japanese population or to societies with different cultural and healthcare systems, this cohort provides valuable insights into community-based health promotion in aging societies. Third, we did not adjust for comorbid conditions, which may confound HRQOL trajectories and affect the observed associations. For example, individuals with cognitive impairment or dementia are less likely to attend routine health-check examinations or may drop out of cohort follow-up entirely, leading to selective attrition that can bias longitudinal estimates^52^. However, only a small number of participants had cognitive impairment; therefore, the impact on the results is likely minimal. Fourth, although there are objective sleep indices such as polysomnography, which is the gold standard for sleep assessment, and actigraphy, which provides a validated and practical alternative for use in non-laboratory settings^53^, this study did not use these objective measures, which limits the interpretation of the associations observed^54^. Nonetheless, the PSQI, while subjective, enabled a feasible and large-scale evaluation of sleep quality in the general population. Future studies should build upon these findings by incorporating objective sleep assessments to elucidate the mechanisms linking sleep and HRQOL more precisely. Finally, the SF-36 and CES-D measures were also scored based on participants’ subjective assessments; therefore, it is important to consider the inherent uncertainty in these scores.

## Conclusion

To the best of our knowledge, this is the first report of the longitudinal HRQOL trajectory among Japanese community-dwelling older adults. The SF-36 subscales exhibited several trajectory patterns, and within the RP and RE trajectory patterns, some patterns showed either a rapid decline or maintained their high scores, despite their similar high baseline scores. To maintain these HRQOL measures related to daily functioning, it is important to ensure improved sleep quality to prevent daytime sleepiness. Our research proposes a future study examining potential strategies for more accurate monitoring approaches and interventions to improve sleep quality (e.g., polysomnography and continuous positive airway pressure therapy) in the general population.

## Supplementary Information

Below is the link to the electronic supplementary material.

Supplementary Material 1

## Data Availability

The dataset analyzed in this study are not publicly available because of ethical concerns; however, accessing confidential data is available upon review and approval by the Hirosaki University COI Program Institutional Data Access/Ethics Committee organization (contact via e-mail: [coi@hirosaki-u.ac.jp]).
