# Supplementary material for: Longitudinal trajectories of health-related quality of life and their predictors among community-dwelling older adults
Source: Sci Rep. 2025 Dec 7;16:872. doi: 10.1038/s41598-025-30307-8 (PMC12783101; doi:10.1038/s41598-025-30307-8)
Supplement: Supplementary file 1 — Supplementary Material 1 [file 41598_2025_30307_MOESM1_ESM.pdf]

Supplementary Information

Longitudinal Trajectories of Health-Related Quality of Life and Their Predictors among Community-Dwelling Older Adults

Ryoga Oshima, BSc [1#], Yuki Ohashi, PhD [1#], Takuro Iwane, BCS [2], Yoshinori Tamada, PhD [3], Fumie Kinoshita, PhD [4], Tomoya Ito, MSc [1], Yuto Okumura, MSc [1], Tatsuya Mikami, MD, PhD [5], Ken Itoh, MD, PhD [6], Koichi Murashita, MSc [2], Masahiro Nakatochi, PhD [1*]

1. Public Health Informatics Unit, Department of Integrated Health Sciences, Nagoya University Graduate School of Medicine, Nagoya, Japan
2. Research Institute of Health Innovation, Hirosaki University, Hirosaki, Japan
3. Department of Medical Data Intelligence, Research Center for Health-Medical Data Science (RCoHMDS), Hirosaki University Graduate School of Medicine, Hirosaki, Japan
4. Department of Advanced Medicine, Data Science Division, Data Coordinating Center, Nagoya University Hospital, Nagoya, Japan
5. Department of Preemptive Medicine, Innovation Center for Health Promotion, Hirosaki University Graduate School of Medicine, Hirosaki, Japan
6. Department of Stress Response Science, Biomedical Research Center, Hirosaki University Graduate School of Medicine, Hirosaki, Japan

# Ryoga Oshima and Yuki Ohashi these two authors contributed equally to this work.

***Correspondence:** Masahiro Nakatochi PhD, Public Health Informatics Unit, Department of Integrated Health Sciences, Nagoya University Graduate School of Medicine, 1-1-20 Daiko-Minami, Higashi-ku, Nagoya, 461-8673, Japan.

E-mail: mnakatochi@met.nagoya-u.ac.jp, Tel: +81-52-719-1923

Supplementary Table S1. Diagnosis of LCMMs with linear, beta, and spline link functions with five knots placed in percentiles.

SF-36 subscale	Link function	AIC	BIC
Physical functioning	LCMM with linear	38441	38480
	**LCMM with beta**	**34753**	**34801**
	LCMM with spline	35967	36030
Role-physical	LCMM with linear	41063	41102
	LCMM with beta*	29275	29323
	**LCMM with spline**	**35824**	**35886**
Bodily pain	LCMM with linear	41613	41651
	**LCMM with beta**	**39085**	**39133**
	LCMM with spline	40457	40519
General health	LCMM with linear	38282	38321
	LCMM with beta	38253	38301
	**LCMM with spline**	**37817**	**37879**
Vitality	LCMM with linear	39768	39807
	**LCMM with beta**	**39369**	**39418**
	LCMM with spline	39592	39655
Social functioning	LCMM with linear	39623	39661
	LCMM with beta*	24332	24380
	**LCMM with spline**	**33346**	**33408**
Role-emotional	LCMM with linear	40464	40502
	LCMM with beta*	25092	25140
	**LCMM with spline**	**33815**	**33878**
Mental health	LCMM with linear	39255	39293
	**LCMM with beta**	**37809**	**37857**
	LCMM with spline	38397	38459

*The maximum absolute gradient exceeded 1.0×10^{-4}, thus failing to meet the convergence criterion. The Akaike information criterion (AIC) and Bayesian information criterion (BIC) provide information on how well each model fits the data, with lower values indicating better model fit. Boldface indicates the best statistical model.

Abbreviation: LCMM, latent class mixed model.

Supplementary Table S2. Model diagnosis and posterior classification data

SF-36 subscale	Latent class number	AIC	BIC	entropy	% in class 1	% in class 2	% in class 3	% in class 4	% in class 5
Physical functioning	1	34753	34801	1.00	100	NA	NA	NA	NA
	**2**	**34745**	**34808**	**0.41**	**28.7**	**71.3**	NA	NA	NA
	3	34736	34813	0.50	29.6	3.6	66.8	NA	NA
	4	34731	34822	0.54	21.6	4.0	55.8	18.6	NA
	5	34731	34837	0.53	3.4	20.3	43.3	27.9	5.1
Role-physical	1	35824	35886	1.00	100	NA	NA	NA	NA
	2	35654	35731	0.78	69.7	30.3	NA	NA	NA
	**3**	**35631**	**35723**	**0.65**	**59.9**	**10.8**	**29.3**	NA	NA
	4	35637	35743	0.45	56.2	13.8	0.0	30.0	NA
	5	35594	35714	0.52	0.0	49.8	19.8	7.9	22.5
Bodily pain	1	39085	39133	1.00	100	NA	NA	NA	NA
	**2**	**39059**	**39122**	**0.55**	**20.0**	**80.0**	NA	NA	NA
	3	39063	39140	0.32	19.1	56.8	24.1	NA	NA
	4	38990	39081	0.74	15.1	18.0	65.9	1.0	NA
	5	38983	39089	0.72	15.5	16.9	64.8	1.8	1.0
General health	**1**	**37817**	**37879**	**1.00**	**100**	NA	NA	NA	NA
	2	37815	37892	0.76	96.5	3.5	NA	NA	NA
	3	37815	37906	0.60	0.3	96.3	3.4	NA	NA
	4	37809	37915	0.66	0.5	5.3	85.3	8.9	NA
	5	37811	37931	0.56	0.8	4.7	24.2	61.6	8.7
Vitality	1	39369	39418	1.00	100	NA	NA	NA	NA
	**2**	**39353**	**39415**	**0.63**	**8.9**	**91.1**	NA	NA	NA
	3	39356	39433	0.36	9.0	50.2	40.8	NA	NA

	4	39352	39443	0.61	8.6	4.8	84.0	2.6	NA
	5	39356	39462	0.57	7.6	6.2	2.3	81.0	3.0
Social functioning	1	33346	33408	1.00	100	NA	NA	NA	NA
	**2**	**33093**	**33170**	**0.86**	**78.7**	**21.3**	NA	NA	NA
	3	33099	33191	0.44	77.7	0.0	22.3	NA	NA
	4	33055	33161	0.44	20.9	59.6	0.0	19.5	NA
	5	33007	33127	0.49	20.7	54.9	0.0	7.4	17.0
Role-emotional	1	33813	33856	1.00	100	NA	NA	NA	NA
	2	33513	33590	0.88	78.8	21.2	NA	NA	NA
	**3**	**33440**	**33531**	**0.76**	**65.7**	**16.1**	**18.1**	NA	NA
	4	33446	33552	0.50	61.8	0.0	18.1	20.1	NA
	5*	34873	33572	0.41	59.5	0.0	0.0	22.4	18.1
Mental health	1	37809	37857	1.00	100	NA	NA	NA	NA
	**2**	**37805**	**37867**	**0.41**	**63.2**	**36.8**	NA	NA	NA
	3	37789	37866	0.50	3.6	58.9	37.5	NA	NA
	4	37791	37882	0.52	3.5	23.7	54.1	18.7	NA
	5	37796	37902	0.44	3.6	17.4	40.4	23.1	15.5

*The maximum absolute gradient exceeded 1.0×10^{-4}, thus failing to meet the convergence criterion. The Akaike information criterion (AIC) and Bayesian information criterion (BIC) provide information on how well each model fits the data, with lower values indicating better model fit. Where a class included less than 5% of the participants, we did not select that number of latent classes, even though the information criterion was the most suitable, and we selected the next best number of classes. Boldface indicates the best statistical model. Abbreviation: NA, not applicable.

Supplementary Table S3. Summary of baseline characteristics by latent classes based on the longitudinal trajectories of the SF-36 role-physical subscale score.

	**Class 1** *N = 545*	**Class 2** *N = 98*	**Class 3** *N = 267*
Age (years)	63.0 (60.0–68.0)	64.5 (60.0–72.0)	65.0 (61.0–72.0)
Men	207 (38.0%)	33 (33.7%)	82 (30.7%)
Body mass index (kg/m^2)	23.1 (21.2–25.0)	23.5 (21.7–25.6)	23.6 (21.7–26.0)
Body fat percentage (%)	26.4 (21.0–31.6)	28.5 (22.4–33.2)	29.3 (22.8–35.3)
Length of education			
>12 years	70 (13.0%)	9 (9.4%)	32 (12.2%)
≤12 years	475 (87.0%)	89 (90.6%)	235 (87.8%)
SF-36 subscale score			
Physical functioning	90.0 (85.0–95.0)	90.0 (75.0–90.0)	70.0 (60.0–85.0)
Role-physical	100.0 (100.0–100.0)	100.0 (93.8–100.0)	68.8 (50.0–81.3)
Bodily pain	84.0 (62.0–100.0)	72.0 (62.0–84.0)	62.0 (42.0–72.0)
General health	62.0 (52.0–77.0)	56.0 (52.0–67.0)	52.0 (45.0–62.0)
Vitality	75.0 (62.5–87.5)	68.8 (56.3–87.5)	56.3 (43.8–68.8)
Social functioning	100.0 (100.0–100.0)	100.0 (87.5–100.0)	87.5 (68.8–100.0)
Role-emotional	100.0 (100.0–100.0)	100.0 (93.8–100.0)	75.0 (50.0–100.0)
Mental health	85.0 (70.0–95.0)	80.0 (65.0–90.0)	70.0 (55.0–85.0)
Drinking habits			
Never or past drinking	324 (59.4%)	67 (67.3%)	183 (68.5%)
Current drinking	221 (40.6%)	32 (32.7%)	84 (31.5%)
Exercise habits			
≥1 time per week	183 (33.6%)	23 (23.5%)	92 (34.5%)
< 1 time per week	362 (66.4%)	75 (76.5%)	175 (65.5%)
Smoking habit			

Never or past smoking	482 (88.4%)	88 (89.8%)	237 (88.8%)
Current smoking	63 (11.6%)	10 (10.2%)	30 (11.2%)
Current marital status			
Yes	434 (78.9%)	73 (73.7%)	196 (71.3%)
No	111 (21.1%)	25 (26.3%)	71 (28.7%)
Family structure	3.0 (2.0–5.0)	3.0 (2.0–5.0)	3.0 (2.0–5.0)
Open-eye one-leg standing (sec)	7.0 (4.2–7.0)	6.5 (2.2–7.0)	5.8 (1.9–7.0)
Grip strength (kg)	28.0 (24.0–37.0)	28.5 (23.6–35.0)	27.0 (23.0–35.0)
MMSE score	29.0 (28.0–30.0)	29.0 (27.0–30.0)	29.0 (28.0–30.0)
CES-D score			
>15 point	37 (7.9%)	10 (11.8%)	80 (33.6%)
≤15 point	508 (92.1%)	88 (88.2%)	187 (66.4%)
Total PSQI score	2.0 (1.0–4.0)	3.0 (1.0–5.0)	4.0 (2.0–6.0)
C1: subjective sleep quality	1.0 (0.0–1.0)	1.0 (0.0–1.0)	1.0 (0.0–1.0)
C2: sleep latency	0.0 (0.0–1.0)	0.0 (0.0–1.0)	1.0 (0.0–1.0)
C3: sleep duration	0.0 (0.0–1.0)	0.0 (0.0–1.0)	0.0 (0.0–1.0)
C4: habitual sleep efficiency	0.0 (0.0–0.0)	0.0 (0.0–0.0)	0.0 (0.0–0.0)
C5: sleep disturbances	1.0 (0.0–1.0)	1.0 (0.0–1.0)	1.0 (0.0–1.0)
C6: use of sleeping medication	0.0 (0.0–0.0)	0.0 (0.0–0.0)	0.0 (0.0–0.0)
C7: daytime dysfunction	0.0 (0.0–0.0)	0.0 (0.0–1.0)	0.0 (0.0–1.0)

Values are presented as median (interquartile range) and count (%). Abbreviations: SF-36, 36-item short-form 36-item health survey; MMSE, mini-mental state examination; CES-D, Centre for Epidemiologic Studies Depression Scale; PSQI, Pittsburgh sleep quality index.

Supplementary Table S4. Summary of baseline characteristics by latent classes based on the longitudinal trajectories of the SF-36 role-emotional subscale score.

	**Class 1**<br>***N = 598***	**Class 2**<br>***N = 147***	**Class 3**<br>***N = 165***
Age (years)	63.0 (60.0–68.0)	65.0 (60.0–71.5)	66.0 (62.0–72.0)
Men	221 (37.0%)	56 (38.1%)	48 (28.4%)
Body mass index (kg/m^2)	23.1 (21.3–25.1)	23.6 (21.8–25.8)	23.4 (21.6–25.2)
Body fat percentage (%)	26.5 (21.2–32.2)	28.1 (23.3–32.2)	30.3 (21.7–35.2)
Length of education			
>12 years	77 (13.0%)	14 (9.7%)	20 (12.4%)
≤12 years	521 (87.0%)	133 (90.3%)	145 (87.6%)
SF-36 subscale score			
Physical functioning	90.0 (80.0–95.0)	85.0 (70.0–95.0)	70.0 (55.0–85.0)
Role-physical	100.0 (93.8–100.0)	93.8 (75.0–100.0)	62.5 (50.0–81.3)
Bodily pain	74.0 (62.0–100.0)	72.0 (57.5–84.0)	62.0 (42.0–72.0)
General health perceptions	62.0 (52.0–76.5)	57.0 (51.0–72.0)	52.0 (42.0–57.0)
Vitality	75.0 (62.5–95.0)	62.5 (50.0–75.0)	50.0 (43.8–62.5)
Social functioning	100.0 (100.0–100.0)	100.0 (81.3–100.0)	75.0 (62.5–100.0)
Role-emotional	100.0 (100.0–100.0)	100.0 (87.5–100.0)	66.7 (50.0–75.0)
Mental health	85.0 (70.0–93.8)	75.0 (60.0–90.0)	65.0 (50.0–80.0)
Drinking habits			
Never or past drinking	365 (61.0%)	93 (63.3%)	115 (69.7%)
Current drinking	233 (39.0%)	54 (36.7%)	50 (30.3%)
Exercise habits			
≥1 time per week	194 (32.4%)	47 (32.0%)	57 (34.5%)
< 1 time per week	404 (67.6%)	100 (68.0%)	108 (65.5%)
Smoking habit			

Never or past smoking	528 (88.3%)	133 (90.5%)	146 (88.5%)
Current smoking	70 (11.7%)	14 (9.5%)	19 (11.5%)
Current marital status			
Yes	474 (78.4%)	117 (78.4%)	112 (65.9%)
No	124 (21.6%)	30 (21.6%)	53 (34.2%)
Family structure	3.0 (2.0–5.0)	3.0 (2.0–5.0)	3.0 (2.0–5.0)
Open-eye one-leg standing (sec)	7.0 (3.9–7.0)	6.9 (2.2–7.0)	5.4 (1.8–7.0)
Grip strength (kg)	28.4 (24.0–36.6)	28.0 (23.5–37.0)	26.0 (23.0–32.6)
MMSE score	29.0 (28.0–30.0)	29.0 (27.0–30.0)	29.0 (27.0–30.0)
CES-D score			
>15 point	37 (7.2%)	21 (15.9%)	69 (47.3%)
≤15 point	561 (92.1%)	126 (84.1%)	96 (52.7%)
Total PSQI score	2.0 (1.0–4.0)	4.0 (2.0–5.0)	4.0 (3.0–7.0)
C1: subjective sleep quality	1.0 (0.0–1.0)	1.0 (0.0–1.0)	1.0 (1.0–1.0)
C2: sleep latency	0.0 (0.0–1.0)	0.0 (0.0–1.0)	1.0 (0.0–2.0)
C3: sleep duration	0.0 (0.0–1.0)	1.0 (0.0–1.0)	0.0 (0.0–1.0)
C4: habitual sleep efficiency	0.0 (0.0–0.0)	0.0 (0.0–0.0)	0.0 (0.0–0.0)
C5: sleep disturbances	1.0 (0.0–1.0)	1.0 (0.0–1.0)	1.0 (1.0–1.0)
C6: use of sleeping medication	0.0 (0.0–0.0)	0.0 (0.0–0.0)	0.0 (0.0–0.0)
C7: daytime dysfunction	0.0 (0.0–0.0)	0.0 (0.0–1.0)	1.0 (0.0–1.0)

Values are presented as median (interquartile range) and count (%). Abbreviations: SF-36, 36-item short-form 36-item health survey; MMSE, mini-mental state examination; CES-D, Centre for Epidemiologic Studies Depression Scale; PSQI, Pittsburgh sleep quality index.

Supplementary Table S5. The additional Firth logistic regression analysis results identifying predictors of the decline and non-decline groups, restricted to SF-36 role-physical/role-emotional subscale score of 100.

	Role-physical			Role-emotional		
	**Complete data n (%)**	**OR (95% CI)**	***p*-value**	**Complete data n (%)**	**OR (95% CI)**	***p*-value**
Body mass index category	498 (100%)			615 (100%)		
Normal range (18.5–25.0 kg/m^2)		1 (reference)			1 (reference)	
Overweight (>25 kg/m^2)		1.61 (0.92–2.77)	0.096		1.85 (1.15–2.94)	0.011
Underweight (≤18.5 kg/m^2)		0.93 (0.24–2.69)	0.900		0.95 (0.29–2.46)	0.923
Body fat percentage	498 (100%)	1.05 (1.00–1.09)	0.030	615 (100%)	1.03 (0.99–1.07)	0.085
Lifestyles						
Presence of drinking habits	497 (99.8%)	0.88 (0.47–1.59)	0.664	614 (99.8%)	0.75 (0.43–1.30)	0.304
Presence of exercise habits	498 (100%)	0.52 (0.28–0.92)	0.024	615 (100%)	0.84 (0.51–1.34)	0.459
Presence of smoking habit	498 (100%)	1.04 (0.43–2.26)	0.934	615 (100%)	0.81 (0.35–1.70)	0.591
Length of education	491 (98.6%)	0.87 (0.36–1.87)	0.737	607 (98.7%)	0.83 (0.38–1.64)	0.611
Household structure						
Marital status	481 (96.6%)	1.09 (0.56–2.03)	0.787	591 (96.1%)	0.76 (0.41–1.35)	0.362
Family structure	497 (99.8%)	1.04 (0.90–1.18)	0.607	614 (99.8%)	1.02 (0.90–1.15)	0.785
Open-eye one-leg standing (10 sec)	433 (86.9%)	0.87 (0.78–0.98)	0.027	526 (85.5%)	0.92 (0.83–1.02)	0.118
Grip strength (kg)	438 (88.0%)	0.98 (0.93–1.04)	0.526	533 (86.7%)	0.99 (0.94–1.04)	0.605
MMSE score	328 (65.9%)	0.90 (0.78–1.03)	0.133	405 (65.9%)	0.97 (0.85–1.11)	0.653
CES-D score	427 (85.7%)	1.14 (0.39–2.77)	0.794	527 (85.7%)	2.78 (1.28–5.74)	0.011
Total PSQI score	490 (98.4%)	1.14 (1.01–1.27)	0.033	603 (98.0%)	1.19 (1.08–1.31)	< 0.001
C1: subjective sleep quality	495 (99.4%)	1.09 (0.71–1.64)	0.695	612 (99.5%)	1.27 (0.88–1.81)	0.196
C2: sleep latency	494 (99.2%)	1.26 (0.91–1.72)	0.153	610 (99.2%)	1.24 (0.93–1.62)	0.134
C3: sleep duration	498 (100%)	1.06 (0.76–1.47)	0.719	615 (100%)	1.30 (0.97–1.74)	0.080

C4: habitual sleep efficiency	498 (100%)	1.02 (0.41–1.87)	0.963	615 (100%)	1.04 (0.48–1.83)	0.905
C5: sleep disturbances	494 (99.2%)	1.24 (0.78–2.00)	0.365	609 (99.0%)	0.99 (0.66–1.49)	0.962
C6: use of sleeping medication	496 (99.6%)	1.14 (0.72–1.69)	0.544	613 (99.7%)	1.77 (1.31–2.41)	< 0.001
C7: daytime dysfunction	495 (99.4%)	2.10 (1.40–3.14)	< 0.001	611 (99.3%)	2.18 (1.50–3.16)	< 0.001

Odds ratios (ORs), 95% confident intervals (CIs), and *p*-values are adjusted for age and sex. Abbreviations: MMSE, mini-mental state examination; CES-D, Centre for Epidemiologic Studies Depression Scale; PSQI, Pittsburgh sleep quality index.

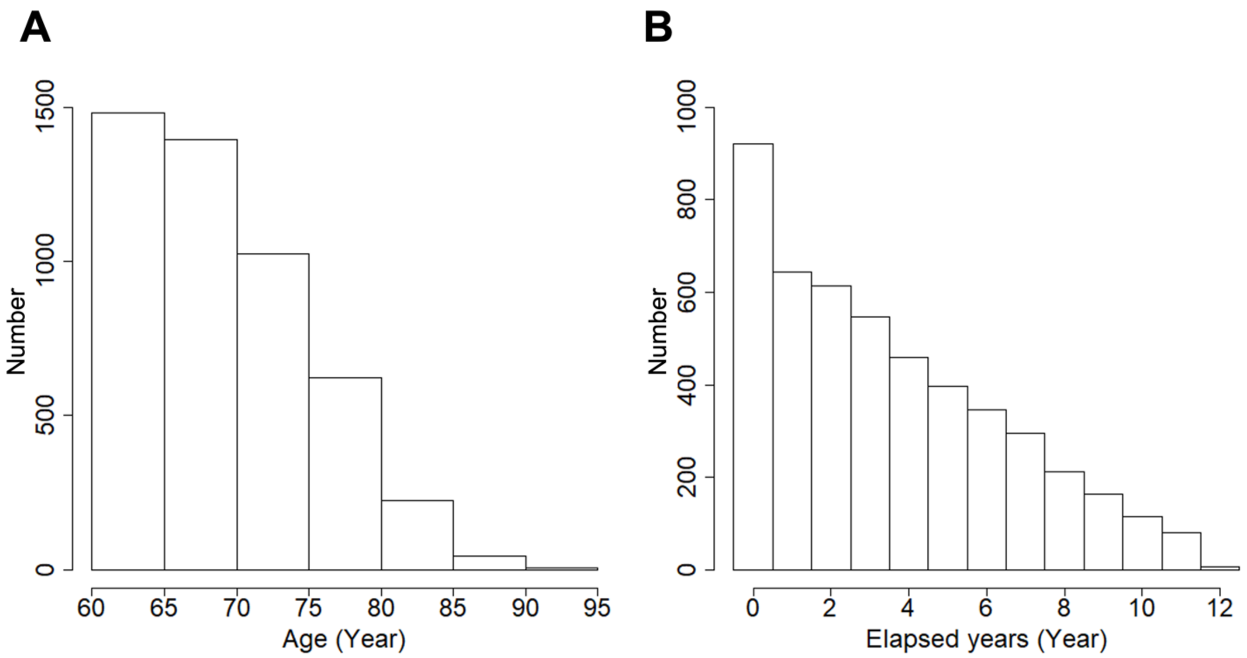

Supplementary Figure S1. Age distribution of all records and participant distribution by elapsed years. These histograms show (a) the age distribution of all records and (b) number of participants by elapsed years since baseline.

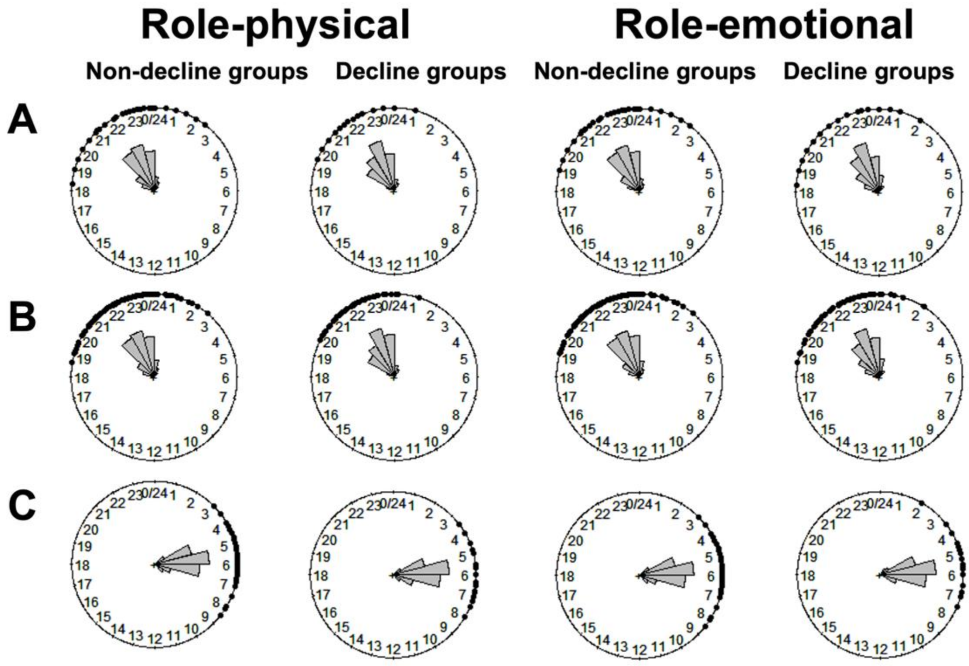

Supplementary Figure S2. Proportion of participants who went to bed, went to sleep, and woke up at each hour of the day. (A) Circular histograms show bedtime, (B) sleep onset time, and (C) waking up time. The grey bars and dots show the proportion of people per hour and the data points, respectively.
